# Supplementary material for: Ruminant-dense environments increase risk of reported Shiga toxin-producing Escherichia coli infections independently of ruminant contact
Source: Appl Environ Microbiol. 2025 Jan 17;91(2):e01864-24. doi: 10.1128/aem.01864-24 (PMC11837525; doi:10.1128/aem.01864-24)
Supplement: Supplemental material — Tables SA1 to SA6. [file aem.01864-24-s0001.docx]

**Appendix Tables**

***Table A1.*** Feedlots and animals on feedlots in Minnesota, 2010-2019.

| Animal Type | Number of Feedlots | Number of Animals | Mean per Feedlot ^a^ |
| --- | --- | --- | --- |
| Cattle | 18,441 | 2,967,940 | 160.9 |
| Swine | 5,869 | 10,611,608 | 1808.1 |
| Goats | 684 | 45,262 | 66.2 |
| Sheep | 1,347 | 206,509 | 153.3 |

^a^ Mean animals per feedlot calculated among feedlots with >0 animals of the given type.

***Table A2.*** Estimated percentages of individuals with contact with a cow, sheep, or goat in the past 7 days by season, age, and sex, calculated from the FoodNet Population Survey *(31)*.

| Season | Age | Male (%) | Female (%) | Both Sexes Combined (%) |
| --- | --- | --- | --- | --- |
| Summer | **0-4** | 14.40 (14.01, 14.81) | 16.05 (8.58, 28.03) | 15.29 (10.83, 21.14) |
|  | **4-9** | 7.49 (5.51, 10.10) | 11.12 (5.74, 20.45) | 9.05 (7.03, 11.57) |
|  | **10-49** | 3.68 (2.46, 5.46) | 12.14 (9.9, 14.81) | 8.22 (6.44, 10.45) |
|  | **50+** | 3.74 (3.22, 4.36) | 5.59 (3.87, 8.01) | 4.69 (3.98, 5.52) |
| Winter | **0-4** | 3.62 (2.23, 5.83) | 6.79 (4.59, 9.93) | 5.03 (3.23, 7.75) |
|  | **4-9** | 3.07 (2.33, 4.06) | 10.10 (6.88, 14.6) | 6.88 (4.92, 9.55) |
|  | **10-49** | 4.35 (3.51, 5.39) | 8.68 (6.64, 11.28) | 6.38 (5.72, 7.12) |
|  | **50+** | 4.73 (3.94, 5.67) | 3.54 (2.73, 4.58) | 4.11 (3.54, 4.77) |

***Table A3.*** Posterior means and 95% credible intervals for incidence rate ratios of STEC infection, combining direct ruminant contact and any reported exposure to a ruminant environment.

| **Summer** | | | | |
| --- | --- | --- | --- | --- |
|  | **With direct ruminant contact only** | | **With direct + indirect ruminant contact** | |
| **Variable** | **O157** | **Non-O157** | **O157** | **Non-O157** |
| **Age** |  |  |  |  |
| 0-4 | 1.00 (Ref) | 1.00 (Ref) | 1.00 (Ref) | 1.00 (Ref) |
| 5-9 | 0.52 (0.41, 0.64)^a^ | 0.41 (0.31, 0.52)^a^ | 0.55 (0.43, 0.68)^a^ | 0.43 (0.33, 0.55)^a^ |
| 10-49 | 0.23(0.20, 0.27)^a^ | 0.32 (0.28, 0.37)^a^ | 0.23 (0.20, 0.27)^a^ | 0.32 (0.28, 0.37)^a^ |
| 50+ | 0.16 (0.13, 0.19)^a^ | 0.12 (0.10, 0.15)^a^ | 0.15 (0.12, 0.18)^a^ | 0.12 (0.09, 0.14)^a^ |
| **Sex** |  |  |  |  |
| Male | 1.00 (Ref) | 1.00 (Ref) | 1.00 (Ref) | 1.00 (Ref) |
| Female | 1.18 (1.04, 1.33)^a^ | 1.54 (1.36, 1.73)^a^ | 1.18 (1.04, 1.33)^a^ | 1.54 (1.36, 1.73)^a^ |
| **Cattle per 10 acres** | 1.30 (1.18, 1.42)^a^ | 0.98 (0.87, 1.11) | 1.29 (1.17, 1.41)^a^ | 0.98 (0.86, 1.10) |
| **Goats per 10 acres** | 2.84 (0.23, 11.96) | 19.51 (1.66, 79.08)^a^ | 2.78 (0.22, 11.70) | 19.32 (1.70, 78.21)^a^ |
| **Sheep per 10 acres** | 2.36 (1.14, 4.17)^a^ | 2.15 (0.95, 4.00)^a^ | 2.35 (1.12, 4.15)^a^ | 2.14 (0.94, 3.99) |
| **Ruminant contact ^b^** |  |  |  |  |
| No | 1.00 (Ref) | 1.00 (Ref) | 1.00 (Ref) | 1.00 (Ref) |
| Yes | 5.11 (4.47, 5.81)^a^ | 7.47 (6.58, 8.42)^a^ | 7.54 (6.61, 8.56)^a^ | 9.61 (8.47, 10.88)^a^ |
| **Winter** | | | | |
|  | **With direct ruminant contact only** | | **With direct + indirect ruminant contact** | |
| **Variable** | **O157** | **Non-O157** | **O157** | **Non-O157** |
| **Age** |  |  |  |  |
| 0-4 | 1.00 (Ref) | 1.00 (Ref) | 1.00 (Ref) | 1.00 (Ref) |
| 5-9 | 0.55 (0.31, 0.87)^a^ | 0.27 (0.16, 0.43)^a^ | 0.56 (0.32, 0.90)^a^ | 0.28 (0.16, 0.44)^a^ |
| 10-49 | 0.50 (0.35, 0.68)^a^ | 0.52 (0.41, 0.66)^a^ | 0.50 (0.35, 0.68)^a^ | 0.52 (0.41, 0.66)^a^ |
| 50+ | 0.40 (0.27, 0.56)^a^ | 0.22 (0.16, 0.29)^a^ | 0.38 (0.27, 0.54)^a^ | 0.22 (0.16, 0.29)^a^ |
| **Sex** |  |  |  |  |
| Male | 1.00 (Ref) | 1.00 (Ref) | 1.00 (Ref) | 1.00 (Ref) |
| Female | 1.67 (1.34, 2.07)^a^ | 1.64 (1.37, 1.96)^a^ | 1.68 (1.34, 2.08)^a^ | 1.64 (1.38, 1.95)^a^ |
| **Cattle per 10 acres** | 0.89 (0.73, 1.07) | 0.98 (0.82, 1.15) | 0.87 (0.71, 1.05) | 0.98 (0.82, 1.15) |
| **Goats per 10 acres** | 20.03 (0.04, 137.85) | 3.79 (0.02, 24.90) | 20.03 (0.04, 137.85)^a^ | 3.86 (0.02, 24.62) |
| **Sheep per 10 acres** | 4.29 (1.42, 8.92)^a^ | 2.59 (0.84, 5.66) | 4.29 (1.42, 8.92)^a^ | 2.59 (0.83, 5.62) |
| **Ruminant contact ^b^** |  |  |  |  |
| No | 1.00 (Ref) | 1.00 (Ref) | 1.00 (Ref) | 1.00 (Ref) |
| Yes | 4.93 (3.76, 6.32)^a^ | 9.01 (7.44, 10.83)^a^ | 6.66 (5.20, 8.40)^a^ | 10.70 (8.87, 12.81)^a^ |

^a^ *p* < 0.05

^b^ Ruminant contact compares cases direct contact to cases with no direct contact for the analyses “with direct ruminant contact only.” For the analyses “with direct + indirect ruminant contact,” the ruminant contact variable compares cases with either direct or indirect contact to cases with neither direct nor indirect contact.

***Table A4.*** Posterior means and 95% credible intervals for incidence rate ratios of STEC infection, including swine density.

| **Summer** | | | | |
| --- | --- | --- | --- | --- |
|  | **Without ruminant contact** | | **With ruminant contact** | |
| **Variable** | **O157** | **Non-O157** | **O157** | **Non-O157** |
| **Age** |  |  |  |  |
| 0-4 | 1.00 (ref) | 1.00 (ref) | 1.00 (ref) | 1.00 (ref) |
| 5-9 | 0.44 (0.35, 0.55) ^a^ | 0.33 (0.26, 0.42) ^a^ | 0.52 (0.41, 0.64) ^a^ | 0.41 (0.31, 0.52) ^a^ |
| 10-49 | 0.24 (0.20, 0.28) ^a^ | 0.33 (0.28, 0.38) ^a^ | 0.23 (0.20, 0.27) ^a^ | 0.32 (0.28, 0.37) ^a^ |
| 50+ | 0.19 (0.16, 0.23) ^a^ | 0.15 (0.13, 0.19) ^a^ | 0.16 (0.13, 0.19) ^a^ | 0.12 (0.10, 0.15) ^a^ |
| **Sex** |  |  |  |  |
| Male | 1.00 (ref) | 1.00 (ref) | 1.00 (ref) | 1.00 (ref) |
| Female | 1.18 (1.04, 1.33) ^a^ | 1.54 (1.36, 1.73) ^a^ | 1.18 (1.04, 1.33) ^a^ | 1.54 (1.36, 1.73) ^a^ |
| **Cattle per 10 acres** | 1.31 (1.19, 1.44) ^a^ | 1.02 (0.90, 1.14) | 1.30 (1.18, 1.42) ^a^ | 0.99 (0.87, 1.11) |
| **Goats per 10 acres** | 3.31 (0.25, 14.32) | 21.22 (1.85, 88.68) ^a^ | 2.86 (0.22, 12.04) | 19.42 (1.67, 79.69) ^a^ |
| **Sheep per 10 acres** | 2.36 (1.14, 4.20) ^a^ | 2.14 (0.94, 3.95) | 2.36 (1.13, 4.20) ^a^ | 2.16 (0.95, 4.01) |
| **Swine per 10 acres** | 1.00 (0.97, 1.03) | 1.00 (0.97, 1.03) | 1.00 (0.97, 1.03) | 1.00 (0.96, 1.03) |
| **Ruminant contact** |  |  |  |  |
| No | 1.00 (ref) | 1.00 (ref) | 1.00 (ref) | 1.00 (ref) |
| Yes | - | - | 5.10 (4.46, 5.81) ^a^ | 7.47 (6.58, 8.45) ^a^ |
| **Winter** | | | | |
|  | **Without ruminant contact** | | **With ruminant contact** | |
| **Variable** | **O157** | **Non-O157** | **Variable** | **O157** |
| **Age** |  |  |  |  |
| 0-4 | 1.00 (ref) | 1.00 (ref) | 1.00 (ref) | 1.00 (ref) |
| 5-9 | 0.50 (0.29, 0.81) ^a^ | 0.23 (0.13, 0.37) ^a^ | 0.55 (0.31, 0.88) ^a^ | 0.27 (0.15, 0.43) ^a^ |
| 10-49 | 0.50 (0.36, 0.69) ^a^ | 0.53 (0.41, 0.68) ^a^ | 0.50 (0.35, 0.68) ^a^ | 0.52 (0.41, 0.67) ^a^ |
| 50+ | 0.46 (0.32, 0.65) ^a^ | 0.28 (0.20, 0.37) ^a^ | 0.40 (0.28, 0.56) ^a^ | 0.22 (0.16, 0.30) ^a^ |
| **Sex** |  |  |  |  |
| Male | 1.00 (ref) | 1.00 (ref) | 1.00 (ref) | 1.00 (ref) |
| Female | 1.67 (1.34, 2.07) ^a^ | 1.64 (1.38, 1.95) ^a^ | 1.68 (1.34, 2.07) ^a^ | 1.65 (1.38, 1.96) ^a^ |
| **Cattle per 10 acres** | 0.97 (0.78, 1.16) | 1.04 (0.87, 1.22) | 0.91 (0.74, 1.10) | 1.00 (0.83, 1.17) |
| **Goats per 10 acres** | 19.42 (0.05, 136.53) | 4.18 (0.03, 26.50) | 18.10 (0.04, 128.20) | 3.72 (0.02, 24.22) |
| **Sheep per 10 acres** | 4.49 (1.45, 9.41) ^a^ | 2.65 (0.86, 5.64) | 4.57 (1.49, 9.62) ^a^ | 2.66 (0.83, 5.75) |
| **Swine per 10 acres** | 0.98 (0.94, 1.03) | 0.98 (0.94, 1.03) | 0.98 (0.94, 1.02) | 0.98 (0.94, 1.03) |
| **Ruminant contact** |  |  |  |  |
| No | 1.00 (ref) | 1.00 (ref) | 1.00 (ref) | 1.00 (ref) |
| Yes | - | - | 4.94 (3.77, 6.31) ^a^ | 9.02 (7.42, 10.82) ^a^ |

^a^ *p* < 0.05

***Table A5.*** Posterior means and 95% credible intervals for incidence rate ratios of O157 STEC infection, using separate models for the time periods 2010-2015 and 2016-2019. The n in each column provides the number of observed O157 STEC cases during that time period.

| **Summer** | | | | |
| --- | --- | --- | --- | --- |
|  | **Without ruminant contact** | | **With ruminant contact** | |
| **Variable** | **2010-2015**  n = 694 | **2016-2019**  n = 363 | **2010-2015**  n = 694 | **2016-2019**  n = 363 |
| **Age** |  |  |  |  |
| 0-4 | 1.00 (Ref) | 1.00 (Ref) | 1.00 (Ref) | 1.00 (Ref) |
| 5-9 | 0.51 (0.39, 0.65) ^a^ | 0.28 (0.16, 0.45) ^a^ | 0.59 (0.45, 0.76) ^a^ | 0.30 (0.18, 0.48) ^a^ |
| 10-49 | 0.20 (0.17, 0.24) ^a^ | 0.32 (0.24, 0.42) ^a^ | 0.20 (0.16, 0.24) ^a^ | 0.32 (0.24, 0.42) ^a^ |
| 50+ | 0.16 (0.13, 0.20) ^a^ | 0.27 (0.19, 0.36) ^a^ | 0.13 (0.11, 0.17) ^a^ | 0.25 (0.18, 0.33) ^a^ |
| **Sex** |  |  |  |  |
| Male | 1.00 (Ref) | 1.00 (Ref) | 1.00 (Ref) | 1.00 (Ref) |
| Female | 1.16 (1.00, 1.35) | 1.22 (0.99, 1.49) | 1.16 (1.00, 1.35) | 1.22 (0.99, 1.49) |
| **Cattle per 10 acres** | 1.35 (1.20, 1.51) ^a^ | 1.27 (1.11, 1.44) ^a^ | 1.34 (1.19, 1.49) ^a^ | 1.27 (1.10, 1.43) ^a^ |
| **Goats per 10 acres** | 3.21 (0.11, 17.23) | 7.71 (0.15, 43.39) | 2.88 (0.10, 15.10) | 7.22 (0.14, 41.12) |
| **Sheep per 10 acres** | 3.56 (1.49, 6.96) ^a^ | 1.49 (0.34, 3.79) | 3.59 (1.50, 6.95) ^a^ | 1.50 (0.35, 3.78) |
| **Ruminant contact** |  |  |  |  |
| No | - | - | 1.00 (Ref) | 1.00 (Ref) |
| Yes | - | - | 4.83 (4.09, 5.65) ^a^ | 2.38 (1.83, 3.02) ^a^ |
| **Winter** | | | | |
|  | **Without ruminant contact** | | **With ruminant contact** | |
| **Variable** | **2010-2015**  n = 163 | **2016-2019**  n = 182 | **2010-2015**  n = 163 | **2016-2019**  n = 182 |
| **Age** |  |  |  |  |
| 0-4 | 1.00 (Ref) | 1.00 (Ref) | 1.00 (Ref) | 1.00 (Ref) |
| 5-9 | 0.50 (0.23, 0.92) ^a^ | 0.53 (0.22, 1.03) | 0.57 (0.26, 1.06) | 0.53 (0.22, 1.05) |
| 10-49 | 0.41 (0.26, 0.64) ^a^ | 0.63 (0.38, 1.02) | 0.41 (0.26, 0.64) ^a^ | 0.63 (0.38, 1.02) |
| 50+ | 0.38 (0.22, 0.60) ^a^ | 0.58 (0.34, 0.96) ^a^ | 0.31 (0.18, 0.50) ^a^ | 0.57 (0.33, 0.93) ^a^ |
| **Sex** |  |  |  |  |
| Male | 1.00 (Ref) | 1.00 (Ref) | 1.00 (Ref) | 1.00 (Ref) |
| Female | 1.64 (1.18, 2.23) ^a^ | 1.73 (1.27, 2.32) ^a^ | 1.64 (1.18, 2.23) ^a^ | 1.73 (1.26, 2.32) ^a^ |
| **Cattle per 10 acres** | 1.11 (0.85, 1.36) | 0.82 (0.59, 1.06) | 1.01 (0.77, 1.28) | 0.82 (0.59, 1.06) |
| **Goats per 10 acres** | 13.88 (0.00, 99.68) | 373.58 (0.06, 2444.03) | 12.24 (0.00, 90.53) | 275.20 (0.06, 2114.03) |
| **Sheep per 10 acres** | 2.82 (0.27, 9.08) | 6.34 (1.64, 14.79) ^a^ | 2.60 (0.25, 8.44) | 6.51 (1.69, 14.67) ^a^ |
| **Ruminant contact** |  |  |  |  |
| No | - | - | 1.00 (Ref) | 1.00 (Ref) |
| Yes | - | - | 7.41 (5.14, 10.28) ^a^ | 1.52 (0.86, 2.41) |

^a^ *p* < 0.05

***Table A6.*** Posterior means and 95% credible intervals for incidence rate ratios of non-O157 STEC infection, using separate models for the time periods 2010-2015 and 2016-2019. The n in each column provides the number of observed non-O157 STEC cases during that time period.

| **Summer** | | | | |
| --- | --- | --- | --- | --- |
|  | **Without ruminant contact** | | **With ruminant contact** | |
| **Variable** | **2010-2015**  n = 537 | **2016-2019**  n = 581 | **2010-2015**  n = 537 | **2016-2019**  n = 581 |
| **Age** |  |  |  |  |
| 0-4 | 1.00 (Ref) | 1.00 (Ref) | 1.00 (Ref) | 1.00 (Ref) |
| 5-9 | 0.45 (0.31, 0.63) ^a^ | 0.25 (0.17, 0.36) ^a^ | 0.56 (0.38, 0.78) ^a^ | 0.28 (0.19, 0.39) ^a^ |
| 10-49 | 0.40 (0.32, 0.50) ^a^ | 0.28 (0.23, 0.34) ^a^ | 0.39 (0.31, 0.49) ^a^ | 0.28 (0.23, 0.34) ^a^ |
| 50+ | 0.16 (0.12, 0.21) ^a^ | 0.15 (0.12, 0.20) ^a^ | 0.12 (0.09, 0.16) ^a^ | 0.13 (0.10, 0.17) ^a^ |
| **Sex** |  |  |  |  |
| Male | 1.00 (Ref) | 1.00 (Ref) | 1.00 (Ref) | 1.00 (Ref) |
| Female | 1.52 (1.28, 1.81) ^a^ | 1.56 (1.32, 1.83) ^a^ | 1.52 (1.28, 1.81) ^a^ | 1.56 (1.32, 1.84) ^a^ |
| **Cattle per 10 acres** | 0.95 (0.79, 1.12) | 1.08 (0.92, 1.24) | 0.91 (0.76, 1.08) | 1.06 (0.91, 1.22) |
| **Goats per 10 acres** | 96.58 (3.21, 512.12) ^a^ | 9.51 (0.16, 54.32) | 94.23 (3.11, 505.97) ^a^ | 8.68 (0.14, 51.65) |
| **Sheep per 10 acres** | 3.12 (0.98, 6.99) | 1.72 (0.51, 3.89) | 3.19 (0.96, 7.17) | 1.72 (0.51, 3.87) |
| **Ruminant contact** |  |  |  |  |
| No | - | - | 1.00 (Ref) | 1.00 (Ref) |
| Yes | - | - | 8.18 (6.80, 9.78) ^a^ | 3.27 (2.69, 3.93) ^a^ |
| **Winter** | | | | |
|  | **Without ruminant contact** | | **With ruminant contact** | |
| **Variable** | **2010-2015**  n = 209 | **2016-2019**  n = 317 | **2010-2015**  n = 209 | **2016-2019**  n = 317 |
| **Age** |  |  |  |  |
| 0-4 | 1.00 (Ref) | 1.00 (Ref) | 1.00 (Ref) | 1.00 (Ref) |
| 5-9 | 0.27 (0.12, 0.49) ^a^ | 0.21 (0.08, 0.40) ^a^ | 0.32 (0.14, 0.59) ^a^ | 0.22 (0.09, 0.43) ^a^ |
| 10-49 | 0.42 (0.29, 0.59) ^a^ | 0.66 (0.46, 0.92) ^a^ | 0.41 (0.28, 0.58) ^a^ | 0.66 (0.46, 0.92) ^a^ |
| 50+ | 0.20 (0.12, 0.31) ^a^ | 0.36 (0.24, 0.53) ^a^ | 0.16 (0.09, 0.24) ^a^ | 0.33 (0.22, 0.48) ^a^ |
| **Sex** |  |  |  |  |
| Male | 1.00 (Ref) | 1.00 (Ref) | 1.00 (Ref) | 1.00 (Ref) |
| Female | 1.67 (1.25, 2.21) ^a^ | 1.64 (1.30, 2.04) ^a^ | 1.68 (1.25, 2.21) ^a^ | 1.64 (1.30, 2.05) ^a^ |
| **Cattle per 10 acres** | 1.02 (0.78, 1.27) | 1.07 (0.87, 1.27) | 0.93 (0.68, 1.20) | 1.04 (0.85, 1.24) |
| **Goats per 10 acres** | 6.26 (0.00, 46.02) | 11.83 (0.04, 79.28) | 6.86 (0.00, 39.83) | 10.63 (0.03, 73.62) |
| **Sheep per 10 acres** | 3.07 (0.42, 9.10) | 2.72 (0.66, 6.56) | 3.00 (0.37, 9.07) | 2.71 (0.66, 6.53) |
| **Ruminant contact** |  |  |  |  |
| No | - | - | 1.00 (Ref) | 1.00 (Ref) |
| Yes | - | - | 11.74 (8.62, 15.53) ^a^ | 3.57 (2.64, 4.68) ^a^ |

^a^ *p* < 0.05
